# The Proposition and Evaluation of the RoEduNet-SIMARGL2021 Network Intrusion Detection Dataset

**DOI:** 10.3390/s21134319

**Published:** 2021-06-24

**Authors:** Maria-Elena Mihailescu, Darius Mihai, Mihai Carabas, Mikołaj Komisarek, Marek Pawlicki, Witold Hołubowicz, Rafał Kozik

**Affiliations:** 1 Department of Computer Science and Engineering, Faculty of Automatic Control and Computer Science, University Politehnica of Bucharest, 060042 Bucharest, Romania; maria.mihailescu@upb.ro (M.-E.M.); darius.mihai@upb.ro (D.M.); 2 RoEduNet, Strada Mendeleev 21-25, 010362 Bucharest, Romania; mihai.carabas@roedu.net; 3 ITTI Sp. z o.o., ul. Rubież 46, 61-612 Poznań, Poland; mikolaj.komisarek@itti.com.pl (M.K.); rafal.kozik@itti.com.pl (R.K.); 4 Institute of Telecommunications and Computer Science, UTP University of Science and Technology, 85-796 Bydgoszcz, Poland; witold.holubowicz@utp.edu.pl

**Keywords:** machine learning, network intrusion detection, dataset

## Abstract

Cybersecurity is an arms race, with both the security and the adversaries attempting to outsmart one another, coming up with new attacks, new ways to defend against those attacks, and again with new ways to circumvent those defences. This situation creates a constant need for novel, realistic cybersecurity datasets. This paper introduces the effects of using machine-learning-based intrusion detection methods in network traffic coming from a real-life architecture. The main contribution of this work is a dataset coming from a real-world, academic network. Real-life traffic was collected and, after performing a series of attacks, a dataset was assembled. The dataset contains 44 network features and an unbalanced distribution of classes. In this work, the capability of the dataset for formulating machine-learning-based models was experimentally evaluated. To investigate the stability of the obtained models, cross-validation was performed, and an array of detection metrics were reported. The gathered dataset is part of an effort to bring security against novel cyberthreats and was completed in the SIMARGL project.

## 1. Introduction

The surge in the number of devices communicating with one another over the Internet is expected to reach 50 billion by the end of the decade [1]. This expansion of the Internet makes network security and cyberthreats a global problem.

Increasingly frequent leaks cause users to lose confidence in whether their data is being kept secure. Furthermore, attacks on critical infrastructure, such as water treatment plants or power stations, can have dire consequences [2,3]. This is why the development of appropriate mechanisms to defend against hackers and malware is crucial. One of the mechanisms at the forefront of attack detection are Intrusion Detection Systems (IDS). The constant evolution of malware drives further development of IDS [4]. One of the most important aspects of state-of-the-art IDS comes with the utilization of the machine-learning (ML) technologies. Apart from the influence of hyperparameter setups [5], these methods are only as good as the data used in the training phase. In cybersecurity, data acquisition is particularly hard. The traffic needs to represent the behavior of realistic and current network architectures and feature contemporary attacks. This, in conjunction with numerous privacy and technological issues, creates a vacuum and a constant need for new cybersecurity datasets. This paper is a preliminary description of the creation of the RoEduNet2021 dataset, along with the initial tests performed with the use of ML benchmark algorithms.

The proposed dataset helps build, train, and evaluate ML algorithms used for intrusion detection, on data which is relevant to contemporary network environment. The characteristics of network traffic change in time; not only are there novel attacks, but the nature of the benign traffic also fluctuates, as new services become popular. Thus, even the datasets which gain popularity in the research community become less and less relevant in time.

The contribution of this work is in the collection of relevant, real-life traffic that will be publicly available, and testing its usability for supervised machine learning methods. On top of that, the dataset, in contrast to other available datasets, contains a set of features which allows for formulation of a machine learning model which can be used for detection in live traffic, a feat not found in other datasets available to researchers.

The work described in this paper is part of the SIMARGL project, which is co-financed by the European Union under the Horizon 2020 program. The main goal of this project is to fight the issue of malware and other novel challenges of cybersecurity. This is achieved by finding new solutions that can effectively deal with the detection and prevention of, among others, network anomalies, stegomalware, ransomware and mobile malware. This research was conducted in conjunction with and the participation of the Romanian Education Network (RoEduNet), a national education and research network in Romania. RoEduNet collected and provided real-life data from its own networks, along with records of attacks. The dataset is geared towards cybersecurity researchers who are interested in examining their methods on contemporary, realistic traffic. The dataset also contains features which can be calculated from live traffic, which makes the dataset highly usable for the construction of deployable network intrusion detection systems.

The paper is structured as follows: in Section 2, the overview of the proposed dataset is introduced, with the sources of traffic, the used attacks and the extracted network features explained. Section 3 enumerates the related works in intrusion detection and recent datasets in the domain. Section 4 contains the description of the proposed methodology, Section 5 encompasses the experiments, and their results and the conclusions are given in Section 6.

## 2. Related Work

The development in the domain of network traffic analysis and the preparation of appropriate techniques for detecting anomalies and threats in computer networks, along with the development of the IDS have resulted in the creation and publication of many research papers. The ML algorithms vary in terms of their sophistication and types of features used [6,7]. The following chapter will examine the state-of-the-art publications from the fields related to network traffic threat detection.

### 2.1. Intrusion Detection Systems

Attacks on networks are becoming more sophisticated and pose a serious threat to various types of infrastructure. Unavailability of services due to various types of attacks drastically reduces the confidence in the security of the stored data.

Systems, such as Intrusion Detection Systems (IDS), are used to defend against unwanted activities. The authors of Reference [8] highlight a cross-section of modern IDS, additionally comparing current datasets used to evaluate the IDS. The paper distinguishes the division of IDS into two categories: signature-based (SIDS—an overview and taxonomy are realized in Reference [9]) and anomaly-based (AIDS—characteristics and examples are presented in Reference [10]).

The literature features a myriad of examples of various types of ID Systems. One example comes from Reference [11], where the authors propose a solution to the problem of attack detection for minority classes. They point out the problem of long learning and detection times of deep neural networks. As a result, they propose a solution that is based on the adaptive synthetic (ADASYN) oversampling [12] and LightGBM (Light Gradient Boosted Machines) [13] technologies. The developers first normalize and encode the original data and then increase the number of samples of minority classes using the data balancing technique, ADASYN. Finally, the data prepared in this way is trained using the LightGBM algorithm. NSL-KDD [14], UNSW-NB15 [15], and CICIDS2017 [16] datasets are used to verify the performance of the proposed solution. This approach results in the precision of 92.57%, 89.56%, and 99.91% in the three test sets, respectively.

The authors of Reference [17] touch on the problem of detecting the zero-day attacks. The authors point out that even systems with frequent data updates are unable to detect zero-day attacks due to the lack of an adequate signature database. Zero-day attacks in their early stages are able to bypass the signature-based network intrusion detection systems. The authors of this paper propose to solve this problem by using RNNIDS, which uses recurrent neural networks (RNNs) to find complex patterns in attacks and create similar samples. Using this approach results in an improved NIDS detection rate.

The IDS developed in Reference [18] presents an approach based on a multi-layer perceptron. The research and testing were realized on the UNSW-NB15 dataset, from which 30 features were selected using the gain factor method. The binarization discretization technique was applied. The model achieved the results of 76.96% accuracy.

Another example comes from Reference [19]. The paper presents the design of an IDS that bases its assumptions of detecting unwanted network traffic on feature selection and ensemble learning. In the first step, the authors eliminate the multidimensionality of the data using the CFS-BA algorithm. The operation of this method is based on selecting the most optimal parameters using feature correlation. The data is then subjected to ensemble learning with two algorithms: Random Forest (RF) and Forest by Penalizing Attributes (Forest PA). The last step of labeling is done by using the voting technique, and, through it, the final decision is made. Testing and learning are carried out on the following datasets: NSL-KDD [14], AWID [20], and CIC-IDS2017 [16]. The experimental results for these datasets oscillate around 99% in terms of accuracy.

### 2.2. Overview of Existing Datasets

Detecting anomalies in the network traffic is a challenging endeavor, as more and more threats enter contemporary networks every day. Many contemporary tools today are based on the aspects of machine learning. The effectiveness of the ML-based methods is directly in proportion to the quality of data on which the model is trained. This is an extremely important activity because it conditions the subsequent correct detection of attacks. In this section, the main features of selected benchmark network datasets (CTU-13 [21], CICIDS 2018 [16], IOT-23, LITNET-2020 [22]) are presented.

#### 2.2.1. CTU-13

In 2011, a team of researchers from the Czech Technical University in Prague created the CTU-13 dataset. Network traffic developers decided to establish a benchmark consisting of anomalies, along with reshuffled background traffic. The entire dataset is composed of thirteen sub-sets representing real network traffic. The set includes over twenty million samples. The distribution of labels in the dataset for each scenario is presented in Table 1. The initial collection was captured in a PCAP file [23]; then, during processing, the unidirectional Netflow traffic was separated and converted to a bidirectional Netflow. With this transformation, more features were obtained, and the client-server traffic was distinguished. The list of features with their descriptions can be found in Table 2.

#### 2.2.2. CICIDS 2018

It was developed by the Canadian Cybersecurity Institute in 2018. The collection contains eight different subsets that represent the collected data over a five-day period. The creators provided within each subset both normal and infected traffic, which contains the following infection types: Brute-force, Heartbleed, Botnet, DoS, DDoS, web attacks, and network infiltration from within. All records were produced using the B-Profile tool to profile abstract human behavioral interactions and produce naturalistic, smooth, background traffic. The collection structure contains 83 features, and it was extracted using the proprietary CICFlowMeter tool. The tool, created by the Canadian Cybersecurity Institute, generates a bidirectional flow of network traffic by determining the direction from source to target and from target to source using the first packet. With this approach, the developers have extracted as many as 83 features, like Duration, Number of packets, Number of bytes, Packet length, etc.

#### 2.2.3. IoT-23

The IoT dataset was entirely developed by the Stratosphere Laboratory in the Czech Republic and was published in 2020. The dataset contains infected and normal traffic and features twenty malicious attacks and three benign captures. The researchers managed to collect the traffic from IoT devices and make it available for developing machine learning algorithms that will effectively defend against these threats. The traffic has been divided into twenty-three scenarios, each containing a different type of malware or attack. The traffic distribution is shown in Table 3. The scheme consists of 20 features and a label. The most active anomalies in this set are PartOfAHorizontalPortScan (213,852,924 samples), Okiru [24] (47,381,241 samples), and DDoS (19,538,713 samples), while the least frequent anomalies are: C&C-Mirai [25] (2 samples), PartOfAHorizontalPortScan-Attack (5 samples), and C&C-HeartBeat-FileDownload (11 samples). The list with the description of all features is shown in Table 4.

#### 2.2.4. LITNET-2020

LITNET-2020, which leveraged an academic network, was published in 2020. The traffic was collected in real-life scenarios. The collection period lasted for ten months, and, during all this time, 12 types of different anomalies were extracted, and the data structure itself contains as many as 85 different features. Table 5 shows the specific number of samples by attack class. In total, the dataset contains 45,492,310 flows.

## 3. Proposition of the RoEduNET2021 Dataset

In this section, the proposition of a dataset that is derived from a real data flow in an academic network is presented. The network data schema is in the Netflow v9 format, and it contains 44 unique features and a label describing each frame. The entire flow contains two different types of DDOS attacks [26] and a PortScan attack [27], in addition to normal traffic. The following subsections will provide descriptions of the network and the overall infrastructure. Furthermore, all the collected features will be presented, and the dataset will be tested using machine learning methods.

### 3.1. Overview of RoEduNet’s Client Infrastructure

**Figure 1:**
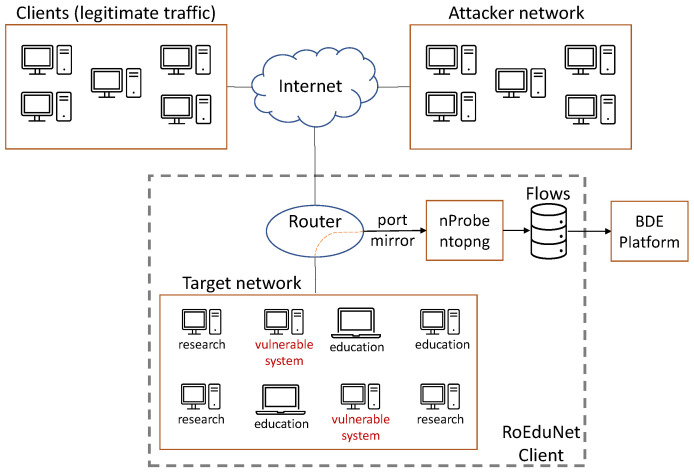
Network architecture for the “Reconnaissance and Denial-of-Service attacks”.

For generating and capturing normal and malicious network traffic, the topology presented in Figure 1 was used:

- Target network: contains the systems that are used in the research laboratories and for educational purposes that are used by one of RoEduNet’s clients. In this network, we added vulnerable systems that will be attacked using different attack scenarios and vectors. This network contains hosts that run Ubuntu, CentOS, or Windows as operating system. The traffic that is generated by the target network (normal and malicious traffic) is represented in dataset.
- Attacker network: contains systems that are used to generate attacks against the vulnerable systems and applications. This network contains virtual machines that run Kali Linux [28] as the operating system. To train the machine learning algorithms, the malicious traffic must be labeled; thus, the source of the attacks must be known. The attacker network is controlled to create and monitor the traffic, and to label the vector attacks.
- Clients (legitimate traffic): represents the traffic flowing through the target network and labeled as normal. Besides malicious traffic, the vulnerable systems contain legitimate traffic, as well.
- Internet: the network architecture is connected to the Internet since the research and education systems highly utilize applications that require Internet connectivity.
- Router: all the previous mentioned network architecture components are converging to the same router. All the traffic that flows through the target network is mirrored, using nProbe and ntopng, and captured.

In the topology presented in Figure 1, the vulnerable systems are the ones that are targeted when running the attacks. Mainly, they contain eLearning platforms (Moodle) that are used in the research and education field. The eLearning platforms were chosen because they are an important part of the university and school activities (especially during online classes) and may represent a target for the entities that want to harm the educational process. The goal is to protect those assets against attacks.

The network used for collecting the data consists of multiple physical and logical elements. The physical elements are the core router, which is comprised of a pair (VSS, Virtual Switching System) of Cisco WS-4506-E with Cisco Catalyst 4500E Supervisor Engine 7-E, to which the the NProbe node has been connected. The core router is also connected to all the switches that bridge the servers from where data of the hosted services is collected. In addition, in this core router, there are multiple links connected to the university campus buildings (from where data generated by RoEduNet’s end-users is collected). The data is collected using Catalyst Switched Port Analyzer (SPAN) from the VLAN interfaces, which are the gateways for the services mentioned above.

NProbe is running on a CentOS7 box that processes the data sent by the SPAN. The services presented in Figure 1 are running on top of an Openstack Cloud deployment. Openstack uses logical links and switches to connect the virtual machines using the Neutron service. The logical links are overlay networks on top of the physical network, implemented using openvswitch.

### 3.2. Traffic and Attack Orchestration

To manage vulnerable servers and to generate legitimate or malicious traffic, virtual machines that are orchestrated using OpenStack [29] were used. OpenStack is an open-source set of tools that can be used to manage a cloud environment.

For the attacker network, a template image was created that is based on Kali Linux that contains the tools necessary for the attacks. In addition, the scripts that can be used to start the attacks were configured. Based on the Kali template, multiple virtual machines were created from where the attacks can be performed.

Even though the attacks are run in a research and education network, and the tests are run during work and classes time, we wanted to have more legitimate traffic. Thus, virtual machines (using OpenStack) were added in the Clients (legitimate traffic) network that use the services alongside with students and researchers. Based on an Ubuntu 20.04 server template, multiple workers, along with a Kubernetes orchestrator [30], were created, that became part of the vulnerable servers’ clients, in addition to students and researchers. Since the platforms used are Moodle instances (eLearning platform), to generate legitimate traffic, JMeter scripts were run to simulate a user’s activity: login, check courses and assignments and logout. The traffic generated by JMeter is not intensive and does not affect the process of generating malicious traffic.

### 3.3. Attack Scenarios

For replicating the real-life use cases, the following attacks were considered to be run into the pilot network: network scanning (reconnaissance) and denial of service.

Usually, network scanning and reconnaissance (commonly implemented using network port scanning) is the first step that is run by an attacker to detect the network connected devices and their configuration details: operating system, open ports, the versions of the running applications and their vulnerability. Thus, one of the attacks that were run against the network is related to network scanning. For running this attack, tools, such as nmap [31] or Masscan [32], were used. For generating network scanning traffic, scanning applications from the attacker network on the IP networks that are contained by the target network are run.

An SYN Scan attack [33] is one the fastest methods of detecting a port’s state. It relies on the TCP three-way handshake where the attacker sends a SYN packet to the desired port. Based on the response (or the lack of it), the attacker can determine if the port is open, closed or has some firewall filters active.

The Denial-of-Service attack category usually leads the system to be inaccessible or to increase the response time to requests. There are multiple methods of attacks that can lead to denial of service. The following two types were chosen:

- Denial-of-Service using SlowLoris [34]: this type of DoS attack opens many HTTP connections to the target and sends incomplete, but legitimate HTTP requests or responses to the target in a very slow manner, keeping the connection alive for a long period of time. Since the HTTP messages are correct and not delivered very fast, they result in flooding the target (as most Denial-of-Service attacks work). The traffic can be considered as legitimate and the attacker as a slow client. Due to the large number of connections that are opened and the slow pace of communication, this type of attack can cause the target to respond very slowly to normal clients, or even to become unresponsive.
- Denial-of-Service using R-U-Dead-Yet (RUDY) [35]: this is also a DoS attack that works in a slow manner to occupy all the target’s processing power by opening and keeping alive many connections and sending responses slowly. However, the main difference between RUDY and SlowLoris is that the first one sends many small HTTP POST messages (usually, 1 byte of data), while the latter sends only HTTP header messages.

For generating Denial-of-Service network traffic, the attacks from the attacker network are started and target the vulnerable servers in the target network.

When the attacks are conducted, the following things need to be taken into consideration to provide a reliable dataset that can be used to train machine learning algorithms:

- The attackers’ IP addresses;
- The targets’ IP addresses;
- The attack’s start and end date.

This information helps properly identify the network packages that should be considered malicious. As shown in Figure 1, using nProbe, all the data that flows through the target network is collected. After the raw logged data is collected and stored (which is saved as a JSON), a Python script is used to convert the logged data into a format that is required by ML algorithms. The script does the following:

- Adds a new key named “LABEL” for each packet. This field specifies if the traffic is considered to be normal (“Normal flow”) or malicious (“SYN Scan”, “Denial of Service SlowLoris”, or “Denial of Service R-U-Dead-Yet”).
- Modifies the key fields from the JSON to match the names described in subsection “Features and labels” (nProbe saves an index for each field, and we replace the index with its name, based on the NetFlow v9/IPFIX format).

### 3.4. Features and Labels

The set of features that was collected from the network infrastructure by the collector and stored in JSON files is based on a data schema in the form of Netflow. This is a network protocol developed by CISCO for collecting and monitoring network flows. During the data collection process, 44 features were extracted that may be needed to correctly analyze network flows and detect anomalies. All the collected featrues are summarized in Table 6.

In addition, each frame contains its own label that specifies exactly the type of flow classifying it as anomalous or not. There are two DDoS attacks (Slowloris and R-U-Dead-Yet) and one PortScan attack type (SYN SCAN) in the dataset. The distribution of these types is as follows: the dataset contains 6,570,058 frames representing the non-infected base traffic. Next, 2,496,814 frames contain the SYN Scan attack. The dataset contains 2,276,947 frames of the Denial of Service R-U-Dead-Yet and Denial of Service Slowloris has 86,4054 flows. In summary, our collection contains 6,570,058 frames of pure traffic and 5,637,815 flows that are labeled as anomalies.

## 4. Proposed Methodology

In this section, the architecture of the created system will be presented. This section also describes the data preparation process detailing all the steps needed to obtain the final version of the schema of the data that will be used later on to prepare and train the model.

### 4.1. Architecture Solution

**Figure 2:**
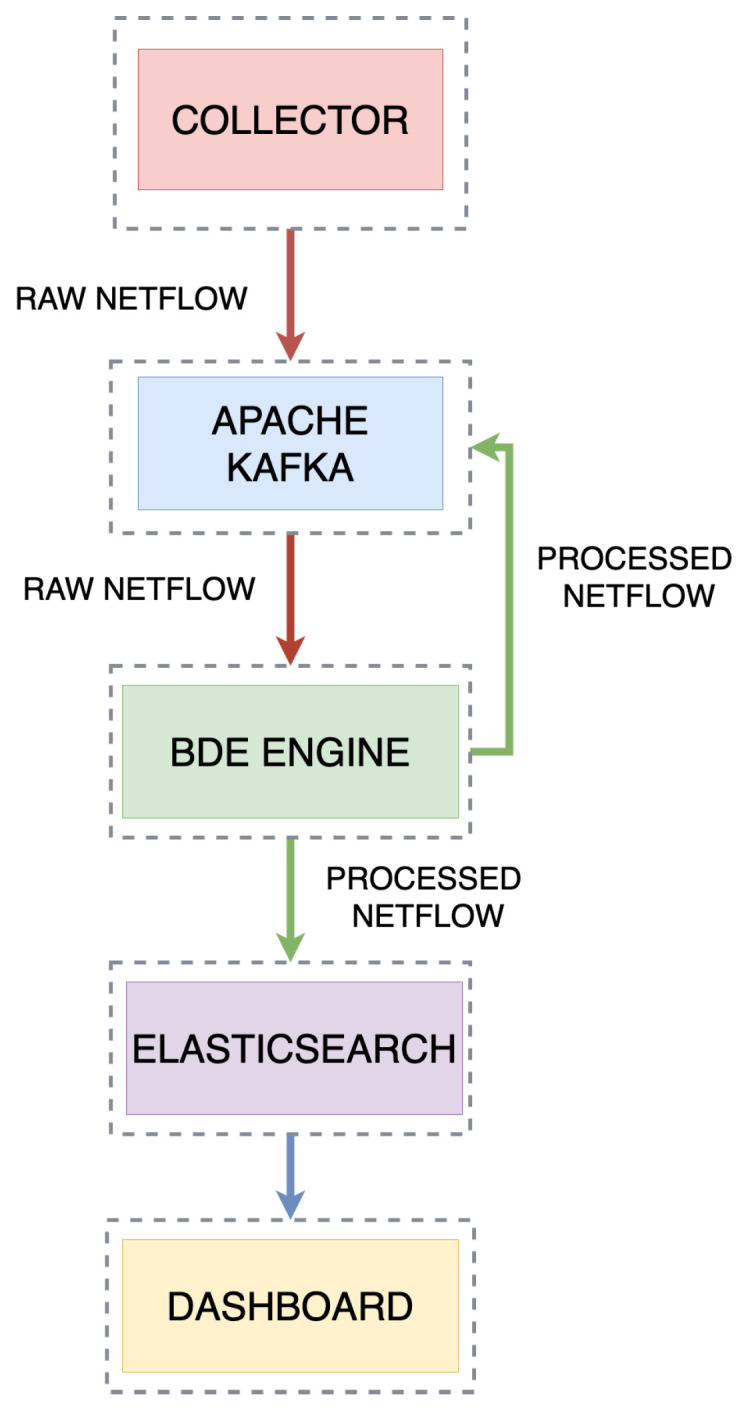
Architecture: Detection Engine.

The process of network intrusion detection occurs in the network environment and can be described by three steps: Collecting Traffic, Delivering Traffic to the Stream, and Verification. Figure 2 shows a simplified diagram of the relationship between the key modules of the system. To properly run real-time stream anomaly detection from the delivered network traffic, it is necessary to train the model in advance. This process is done offline and an initial collection of labeled data is required. Once the data model is created and stored, one can move to the next step which is to perform the live detection. The entire process starts with collecting data and delivering it to the Kafka [36] stream. The detector is set up to work with network data that is delivered in Netflow version 9 format. The detector is developed to work in a real-life situation of real-time network intrusion detection, where the traffic from an environment will be collected with a probe, like *NTOPNG* [37], which provides the ability to collect and transport network traffic to the stream in any form.

The use of the Apache Kafka software in the detection environment is dictated by a number of necessities of real-time network intrusion detection. These are, among others, providing high throughput and low latency message queuing services. Kafka uses the Publish and Subscribe message handling model and stores partitioned data streams securely in a distributed, replicated cluster. Kafka scales linearly as throughput increases.

The traffic delivered to the Kafka stream is received in real time by the detection engine. All features of a single frame are prepared for verification by a suite of machine learning algorithms. After passing through the verification system, the frame is assigned a label. Clean traffic which does not bear any signatures of an attack is labeled as “Normal Flow”. Infected traffic receives a specific label corresponding to the attack type. The intricacies of the stream-based network intrusion detection have been presented in Reference [38]. The final step is to prepare the tagged frame for sending to the Elasticsearch database.

### 4.2. Data Preparation

**Figure 3:**
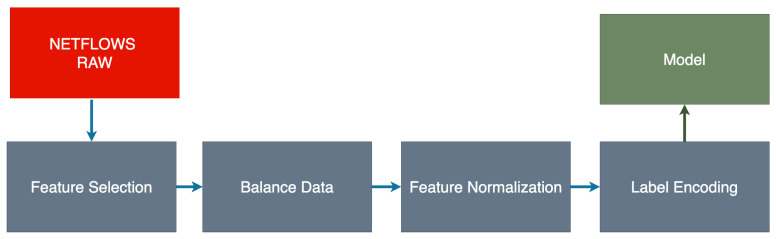
Process of preparing the collected data.

The data preparation process is a crucial step in the ML pipeline. The data preparation steps performed are presented in Figure 3. The listed elements of the data preparation process are described below.

### 4.3. Feature Selection

The first step in the process of preparing the final data shape is feature selection. As the name suggests, feature selection is about choosing from among all features only those that contribute to the effectiveness of the model. Feature selection reduces the computational cost, as well as, in many cases, improves the model performance [39]. Feature selection methods evaluate the relationship between each input variable and the target variable.

For this research, the SelectKBest method was used for feature selection, with the result function set to *chi2*. The Chi-Square method allows for determination of whether the occurrence of a particular trait and the occurrence of a particular class are independent. This can be expressed by the following formula:

(1) $$chi^{2}=\sum_{e_{t}\in \left\{0,1\right\}}\sum_{e_{c}\in \left\{0,1\right\}}\frac{{\left(N_{e_{t}e_{c}}-E_{e_{t}e_{c}}\right)}^{2}}{E_{e_{t}e_{c}}}.$$

*N* is the observed value of w, and *E* the expected value. $e_{t}$ takes the value of 1 if the document contains the term *t*, and 0 otherwise. $e_{c}$ takes the value 1 if the document belongs to class *c*, and 0 otherwise.

**Figure 4:**
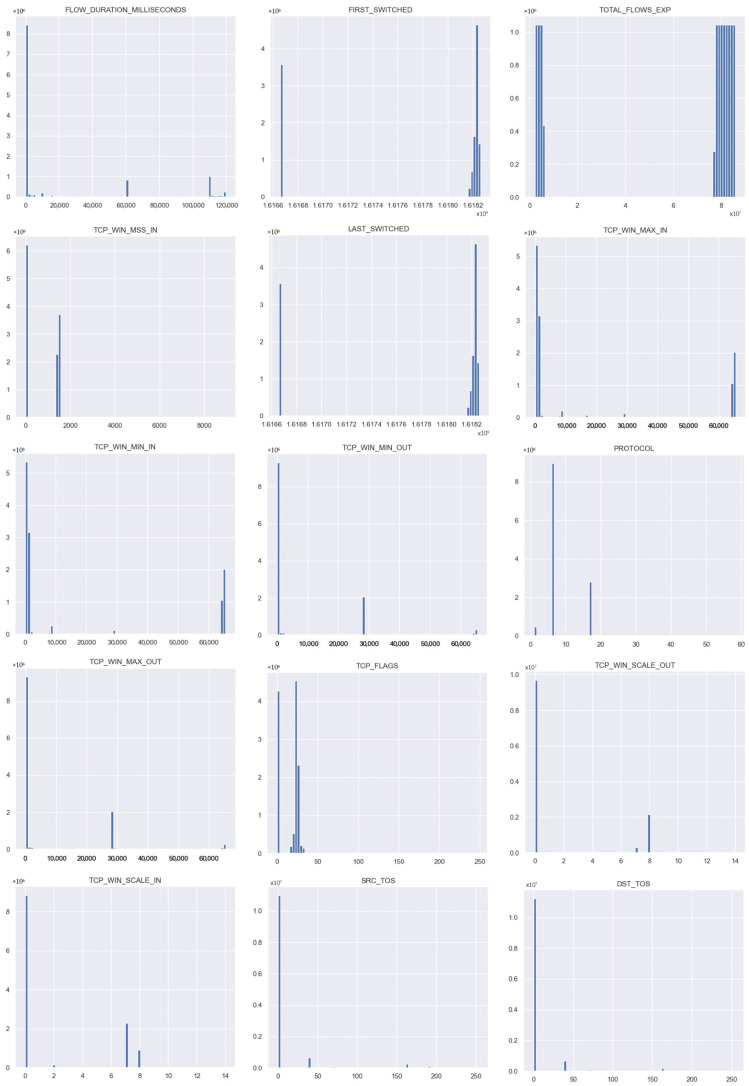
Distribution plots of the 15 most important features.

Each feature in the dataset that receives a high Chi-Square score should be discarded as it means that the class has no effect on the incidence of the feature. Conversely, when the score value is low, it means that the class and the feature are dependent. In Figure 4, the distributions of the 15 most important features in the dataset is shown.

### 4.4. Resolving the Data Imbalance Problem

Uneven distribution of classes is a known ML challenge [40,41,42]. Many ML algorithms can under-perform on imbalanced data, experiencing issues, like misclassification of samples from minority classes to majority classes.

To solve the imbalance issue, SMOTE technique was used at the data preparation stage. SMOTE is one of the most commonly used oversampling methods. This technique was first defined and presented in Reference [43]. It aims to balance the class distribution by increasing the minority class instances with the use of an adaptation of the nearest neighbors algorithm.

To create a synthetic instance, it finds the K-nearest neighbors of each minority sample, randomly selecting one of them, and then computes linear interpolations to create a new minority sample.

For this research, one of the extensions of SMOTE, SMOTE-ENC (Encoded Nominal and Continuous Synthetic Minority Oversampling Techinque), was used. The reason for choosing this particular method was that the data schema contained categorical values. The authors of Reference [44] show the correct results of using this method on categorical values and confirm that this method works correctly. In SMOTE-ENC, if the sample of a categorical attribute differs from its nearest neighbors, then a constant value is added during distance calculation. This method allows for the use of SMOTE on datasets containing both continuous and categorical features.

### 4.5. Feature Standardization

After the dataset was balanced, all samples were subjected to the standardization process. The values were standardized by removing the mean and scaling to unit variance with the use of scikit-learn StandardScaler. To maintain consistency in our tests, we have also centered and scaled the features for the decision-based methods, even though RandomForest can handle both scaled and unscaled features.

### 4.6. Label Encoding

Categorical and textual data is a fairly common occurrence in datasets. In our case, fields, such as protocol and label, are precisely of the categorical type. Some ML algorithms can handle categorical features, but most expect only numeric values. Therefore, all categorical values in the dataset are converted to numeric values. There are multiple ways to perform this conversion; in this work, two were used: One-Hot-Encoding and Label-Encoding. The Label-Encoder method converts each value in the column to a number assigning a value according to the order of appearance, and it is suitable for conversion of the dependent variable. The second approach creates a new column for each category and fills it with zeros (False), only assigning ones (True) for samples where the particular value of the feature was present, making it suitable to use on features.

## 5. Experiments and Results

This section describes and details the tests and provides the results of the study. The formulas by which the machine learning and neural network algorithms were tested and compared on the dataset are specified.

### 5.1. Evaluation Metrics

In this paper, a standard set of well-known metrics was used to evaluate the approach: Accuracy (ACC), Precision (Pr), Recall (Re), F1-Score, Matthews correlation coefficient (MCC) [45], and Balanced accuracy (BCC).

The metrics are calculated with the use of the confusion matrix. The following are the values featured in the confusion matrix: True Positives (TP), which specify correctly predicted positive values, followed by True Negatives (TN), which are correctly predicted negative values. The other two variables are described as False Positives (FP), which is when the result of the actual class is false and the result of the predicted class is positive. The last variable is False Negatives (FN), which is when the actual class is positively classified, but the predicted class indicates a negative value. Presented below are the individual formulas that were considered in the process of evaluating the performance of the algorithm.

$Accuracy=\frac{TP+TN}{TP+FP+FN+TN}$;
$Precision=\frac{TP}{TP+FP}$;
$Recall=\frac{TP}{TP+FN}$;
$F1=2*\frac{Recall*Precision}{Recall+Precision}$;
$BACC=\frac{\frac{TP}{TP+FN}+\frac{TN}{TN+FP}}{2}$;
$MCC=\frac{TN*TP-FN*FP}{\sqrt{\left(TP+FP)(TP+FN)(TN+FP)(TN+FN\right)}}$.

### 5.2. Results

**Figure 5:**
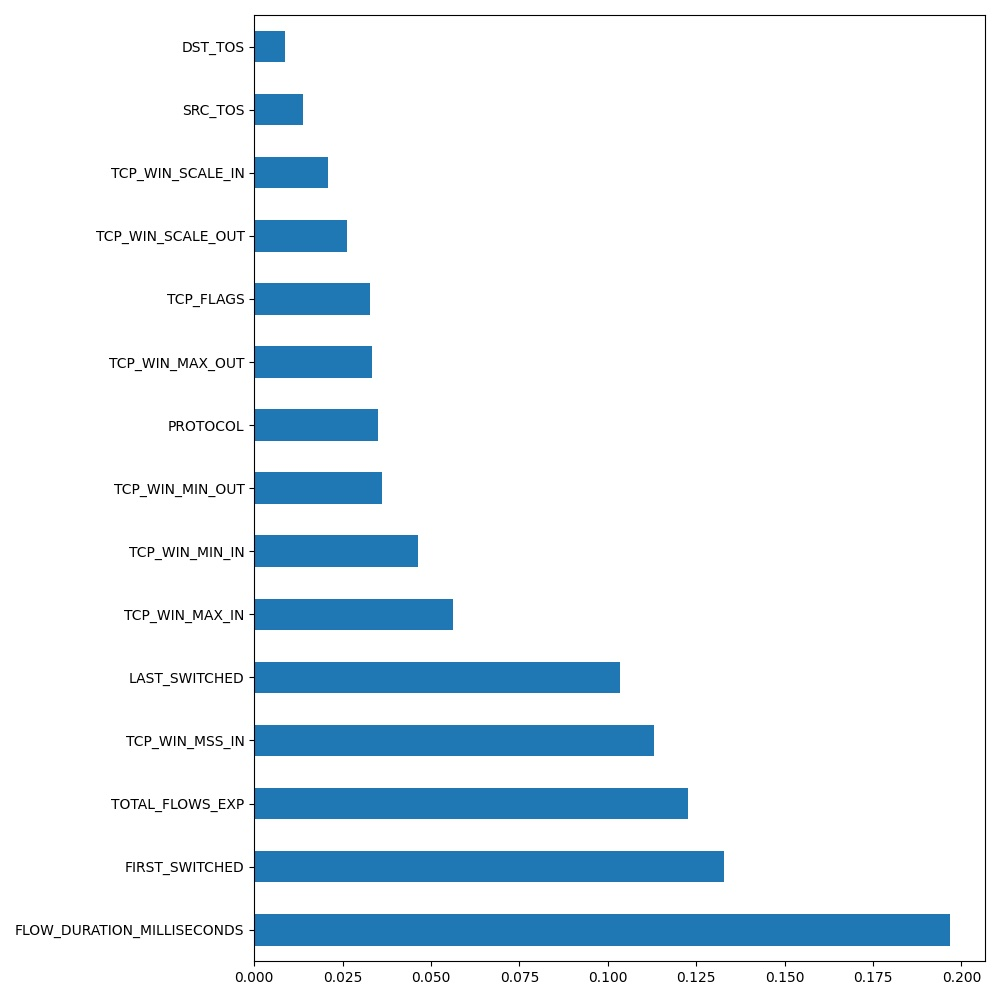
Result of feature selection.

In this section, the results that were achieved after detecting the malicious traffic in the dataset that was collected from an academic network are presented. At the very beginning, the data schema that was provided in Section 3.4 was subjected to feature selection, and the 15 most useful features from this dataset that have the greatest impact on the effectiveness of the model were extracted. A summary of these features can be found in Figure 5. The Y-axis features the 15 parameter names with the highest score.

The feature with the strongest influence on the result of the classification, according to the SelectKBest method [46], is the duration of the data flow.

In the remaining part of the research the focus was on utilizing the following ML methods: Deep neural network [47,48,49], the Random Forest Classifier [50,51], the AdaBoost Classifier [52], and the Gradient Boosted Trees Classifier [53].

The choice of these algorithms was dictated by the following factors: Random Forest has been proven in multiple studies on network attacks; its performance was always high [4], and results were satisfactory, and the authors have found promising results from the utilization of this algorithm in earlier work [54,55]. The Gradient Boosted Trees (GBT) algorithm combines the advantages of RandomForest with the added benefit of gradient utilization. Artificial neural networks were used because they have been proven to continue to learn even when the other methods reach their full potential. Thus, adding ANNs can be a good opportunity to improve results with larger amounts of data. The AdaBoost algorithm was selected to check its potential in the real-world implementation of the NIDS component in the SIMARGL project: the algorithm is fast, simple to use, and does not need extensive hyperparameter tuning.

The selection of hyperparameters in the used algorithms was done using gridSearch, which performs an exhaustive search over the chosen hyperparameter space. The setting of hyperparameters can be a decisive factor for the results obtained by machine-learning methods, as was presented in Reference [5].

Each classifier was subjected to a learning procedure on a training set. Cross-validation was used to test and evaluate the model more accurately. This is a procedure that is used to resample the data. The number of groups into which the set is divided is defined using the K parameter was set, in this case, to 10. Therefore, each result in the summary table of a given test contains 10 records. Each classifier underwent the learning procedure on the training set.

The first classifier is a deep neural network. The architecture of this classifier consists of an input layer with the count of neurons corresponding to the used number of features, and the Rectified Linear Unit (ReLU) activation function. This is followed by a dropout layer with the dropout set at 0.01, and another hidden layer with a set of 16 neurons and the ReLU activation function. The setup closes with a “softmax”. The loss function was set to the “categorical_crossentropy” method, while the chosen optimization algorithm was Adaptive Momentum (ADAM) [56]. Eleven epochs were needed to train the model with a batch size of five. The test results for this model can be found in Table 7.

The next classifier that was used to detect malicious samples in the network traffic was the RandomForest. The settings of this classifier were as follows: n_estimators was set to the value of 100, and this parameter signifies the number of trees used. The maximum tree depth was set to the value of ten, the minimum number of samples required to separate the internal node was set to the value of two. The rest of the settings were used as provided by default. Test results for this model can be found in Table 8.

The Gradient Boosted Trees classifier, is another model that was selected for test-running the dataset. The preparation of this classifier consisted of setting the learning rate to 0.5, the number of boosting steps to be performed was set to 100, the fractions of samples was set to 0.5, the maximum depth of each regression estimator was set to 2 and the number of features to be considered in the search for the best split was set to 2. Test results for this model can be found in Table 9.

The last classifier that was utilized in the study was AdaBoost. The parameter configuration of this classifier was as follows: The maximum number of estimators at which boosting will be completed was set to a value of 50. The weight applied to each classifier in each boosting iteration was set to a value of 1, and the base estimator from which the boosted ensemble is built was set to DecisionTreeClassifier. Test results for this model can be found in Table 10.

To further summarize the results of the experiment the measured metrics for all the used algorithms are gathered in Table 11. The random forest classifier has achieved the best metrics. For comparison, the results of the classifiers without the SMOTE data balancing applied are provided in Table 12. The results are given on a 60/40 train/test split.

In order to compare the different classifiers on the accuracy of each model, a statistical method based on paired Wilcoxon test [57] was applied. The results of these tests are presented in Table 13. It can be seen that the AdaBoost algorithm loses every time compared to all others, while the best choice is DNN or GBT algorithm, whose results are comparable.

## 6. Conclusions

The work presented in this paper provides the results of efficient detection of anomalies in network traffic coming from a real-life architecture. As part of the presented research, traffic from a real-world academic network was collected and, after performing a series of attacks, a dataset was formed. The dataset contains 44 network features and an unbalanced distribution of classes. The traffic captures were annotated accordingly. The efficacy of the dataset for training machine learning algorithms was experimentally evaluated. To investigate the stability of the obtained ML models, cross-validation was performed, and a series of standard detection metrics were reported. The utility of the obtained dataset has been evaluated for the following ML algorithms: Random Forest Classifier, Gradient Boosting Classifier, and a Neural Network. The obtained dataset is part of an ongoing endeavor to provide security against novel cyberthreats, executed in the SIMARGL project.

Although the proposed infrastructure generates attacks, collects, and labels the traffic, it can be improved. The current approach is to generate one attack at a time. However, in a real life environment, multiple attacks may be simultaneously run to destabilize various services: DNS, email, e-learning platforms. Thus, as future work, more complex scenarios may help the researchers train their machine learning algorithms using datasets that are even closer to real-life network traffic.

Moreover, due to physical resource limitations, the proposed infrastructure does not scale well, since larger amounts of data cannot be generated without affecting the functionality of RoEduNet’s client network. The most important limitations that were encountered are the limited disk storage for logged data collected by NProbe, traffic generated through port mirroring sent to NProbe to process data, or large datasets that must be manually transferred from the source to the BDE Platform. Thus, in the future, a more scalable infrastructure should be implemented, as well as an integration procedure that delivers data directly from the source to the BDE Platform.

In the current implementation, the attacks that are generated must be manually started and stopped at well established moments (each attack runs in a well defined time interval so that the traffic can be labeled accordingly). A further improvement that should be added would be to automatically run and label attacks based on a given schedule.

For future work, more different types of attacks are going to be added to the dataset. The number and variety of normal traffic samples are also going to be increased. In addition, this collection is set to become publicly available to provide more researchers the ability to test improve their cybersecurity solutions on contemporary and realistic traffic. In scope of the SIMARGL project, the aim is to provide RoEduNet with a NIDS solution to suit their needs. In addition, future work is dedicated to further improvements towards integrating more machine learning concepts and algorithms, including the notion of online learning, lifelong learning, and unsupervised anomaly detection.

## Figures and Tables

**Table 1:** Distribution of network traffic in the CTU-13 dataset for each scenario.

#	Normal Flows	Background Flows	C&C Flows	Botnet Flows
1	30,387	2,753,290	1026	39,993
2	9120	1,778,061	2102	18,839
3	116,887	4,566,929	63	26,759
4	25,268	1,094,040	49	1719
5	4679	124,252	206	695
6	7494	546,795	199	4431
7	1677	112,337	26	37
8	72,822	2,875,282	1074	5052
9	43,340	2,525,565	5099	179,880
10	15,847	1,187,592	37	106,315
11	2718	96,369	3	8161
12	7628	315,675	25	2143
13	31,791	1,853,217	1202	38,791

**Table 2:** CTU-13 list of features.

Feature Name	Description
StartTime	Start Time flow
SrcAddr	Source IP address
Sport	Source port
DstAddr	Destination IP address
Dport	Destination port
Proto	Protocol
Dir	Direction of communication
Dur	Flow total duration
State	Protocol state
sTos	Source Type of Service
dTos	Destination Type of Service
TotPkts	Total number of packets exchanged
TotBytes	Total number of bytes exchanged
SrcBytes	Number of bytes from source
Label	Name of type attack

**Table 3:** Representation of the traffic content of the IoT-23 dataset by executed attacks.

Attack Name	Flows
Part-Of-A-Horizontal-PortScan	213,852,924
Okiru	47,381,241
Okiru-Attack	13,609,479
DDoS	19,538,713
C&C-Heart Beat	33,673
C&C	21,995
Attack	9398
C&C-	888
C&C-Heart Beat Attack	883
C&C-File download	53
C&C-Tori	30
File download	18
C&C-Heart Beat File Download	11
Part-Of-A-Horizontal-PortScan Attack	5
C&C-Mirai	2

**Table 4:** IOT-23 features.

Feature Name	Description
fields-ts	Start Time flow
uid	Unique ID
id.orig-h	Source IP address
id.orig-p	Source port
id.resp-h	Destination IP address
id.resp-p	Destination port
proto	Protocol
service	Type of Service (http, dns, etc.)
duration	Flow total duration
orig-bytes	Source—destination transaction bytes
resp-bytes	Destination—source transaction bytes
conn-state	Connection state
local-orig	Source local address
local-resp	Destination local address
resp-pkts	Destination packets
orig-ip-bytes	Flow of source bytes
history orig-pkts	History of source packets
missed-bytes	Missing bytes during transaction
tunnel-parents	Traffic tunnel
resp-ip-bytes	Flow of destination bytes
label	Name of type attack

**Table 5:** Representation of the traffic content of the LITNET-2020 dataset by executed attacks.

Attack Name	Number of Samples	Attacks
Packet Fragmentation attack	1,244,866	477
Scanning/spread	6687	6232
Reaper worm	4,377,656	1176
Spam bot’s detection	1,153,020	747
Code red worm	5,082,952	1,255,702
Blaster worm	2,858,573	24,291
LANDattack	3,569,838	52,417
HTTP-flood	3,963,168	22,959
TCP SYN-flood	14,608,678	3,725,838
UDP-flood	606,814	59,479
ICMP-flood	3,863,655	11,628
Smurf	3,994,426	59,479

**Table 6:** The list of the collected network features.

Feature	Description
BIFLOW_DIRECTION	Determines who initiated the flow
DIRECTION	Determines the direction of flow
DST_TO_SRC_SECOND_BYTES	An indicator that determines the flow of bytes per
	second (dst to src)
FIREWALL_EVEN	Information flag from the firewall
FIRST_SWITCHED	Time of appearance of the first flow
FLOW_ACTIVE_TIMEOUT	Network traffic activity timeout
FLOW_DURATION_MICROSECONDS	Duration of flow expressed in microseconds
FLOW_DURATION_MILLISECONDS	Duration of flow expressed in milliseconds
FLOW_END_MILLISECONDS	Duration of flow end expressed in milliseconds
FLOW_END_SEC	Duration of flow end expressed in seconds
FLOW_ID	Unique ID
FLOW_INACTIVE_TIMEOUT	Inactivity time of the flow
FLOW_START_MILLISECONDS	Duration of flow start expressed in milliseconds
FLOW_START_SEC	Duration of flow start expressed in seconds
FRAME_LENGTH	Frame length
IN_BYTES	Number of incoming bytes
IN_PKTS	Number of incoming packets
IPV4_DST_ADDR	Destination IP V4 address
IPV4_SRC_ADDR	Source IP V4 address
L4_DST_PORT	Destination Port
L4_SRC_PORT	Source Port
LAST_SWITCHED	Time of the last packet
MAX_IP_PKT_LEN	The largest length of the observed packet
MIN_IP_PKT_LEN	The smallest length of the observed packet
OOORDER_IN_PKTS	Number of incoming packets that were out of order
OOORDER_OUT_PKTS	Number of outgoing packets that were out of order
OUT_BYTES	Outgoing bytes
OUT_PKTS	Outgoing packets
PROTOCOL	Protocol flag
PROTOCOL_MAP	Name of protocol
RETRANSMITTED_IN_BYTES	Number of incoming bytes repeated
RETRANSMITTED_IN_PKTS	Number of incoming packets repeated
RETRANSMITTED_OUT_BYTES	Number of outgoing bytes repeated
RETRANSMITTED_OUT_PKTS	Number of outgoing packets repeated
SRC_TO_DST_SECOND_BYTES	An indicator that determines the flow of bytes per
	second (src to dst)
TCP_WIN_MAX_IN	Maximum incoming TCP window size
TCP_WIN_MAX_OUT	Maximum outgoing TCP window size
TCP_WIN_MIN_IN	Minimum incoming TCP window size
TCP_WIN_MIN_OUT	Minimum outgoing TCP window size
TCP_WIN_MSS_IN	Incoming TCP segment size
TCP_WIN_MSS_OUT	Outgoing TCP segment size
TCP_WIN_SCALE_IN	Incoming TCP scale size
SRC_TOS	Sets the service type byte on entry to
	the incoming interface.
L7_PROTO_NAME	Name of the layer 7 protocol
TOTAL_FLOWS_EXP	Total number of exported flows

**Table 7:** Summary of the results for the deep neural network.

#	Accuracy	Precision	Recall	F1	BACC	MCC	AUC_ROC
1	0.99	0.99	0.99	0.99	0.9904	0.9873	0.9987
2	0.99	0.99	0.99	0.99	0.9935	0.9914	0.9989
3	0.99	0.99	0.99	0.99	0.9930	0.9908	0.9966
4	0.99	0.99	0.99	0.99	0.9932	0.9910	0.9986
5	1	1	1	1	0.9955	0.9910	0.9983
6	0.99	0.99	0.99	0.99	0.9949	0.9932	0.9987
7	1	1	1	1	0.9953	0.9938	0.9982
8	1	1	1	1	0.9969	0.9959	0.9985
9	0.99	0.99	0.99	0.99	0.9906	0.9874	0.9975
10	0.99	0.99	0.99	0.99	0.9930	0.9907	0.9985

**Table 8:** Summary of the results for the Random Forest Classifier.

#	Accuracy	Precision	Recall	F1	BACC	MCC	AUC_ROC
1	1	1	1	1	0.9999	0.9999	0.9999
2	1	1	1	1	0.9999	0.9999	0.9999
3	1	1	1	1	0.9999	0.9999	0.9999
4	1	1	1	1	0.9999	0.9999	0.9999
5	0.85	0.91	0.85	0.84	0.8537	0.8290	0.9025
6	1	1	1	1	0.9999	0.9999	0.9999
7	0.87	0.91	0.87	0.86	0.8709	0.8469	0.9139
8	1	1	1	1	0.9999	0.9999	0.9997
9	0.85	0.91	0.85	0.84	0.8524	0.8276	0.9016
10	0.78	0.88	0.78	0.72	0.7772	0.7546	0.8514

**Table 9:** Summary of the results for the Gradient Boosting Classifier.

#	Accuracy	Precision	Recall	F1	BACC	MCC	AUC_ROC
1	1	1	1	1	0.9991	0.9988	0.9991
2	1	1	1	1	0.9990	0.9987	0.9993
3	1	1	1	1	0.9993	0.9991	0.9993
4	1	0.99	1	1	0.9985	0.9980	0.9993
5	0.97	0.97	0.97	0.97	0.9660	0.9560	0.9992
6	0.98	0.99	0.98	0.98	0.9849	0.9801	0.9993
7	0.99	0.99	0.99	0.99	0.9927	0.9904	0.9994
8	1	1	1	1	0.9978	0.9971	0.9995
9	0.95	0.96	0.95	0.95	0.9520	0.9388	0.9991
10	0.98	0.98	0.98	0.98	0.9841	0.9791	0.9993

**Table 10:** Summary of the results for the AdaBoost Classifier.

#	Accuracy	Precision	Recall	F1	BACC	MCC	AUC_ROC
1	0.52	0.42	0.53	0.46	0.5214	0.3919	0.7092
2	0.53	0.41	0.52	0.45	0.5313	0.4051	0.7093
3	0.53	0.54	0.52	0.69	0.5332	0.4070	0.7093
4	0.55	0.42	0.53	0.46	0.5459	0.4239	0.7095
5	0.54	0.43	0.54	0.47	0.5313	0.4051	0.7096
6	0.53	0.42	0.53	0.46	0.5332	0.4070	0.7092
7	0.55	0.43	0.55	0.48	0.5459	0.4239	0.7097
8	0.54	0.43	0.54	0.47	0.5423	0.4190	0.7095
9	0.52	0.40	0.52	0.44	0.5244	0.3965	0.7093
10	0.78	0.88	0.78	0.72	0.7762	0.7535	0.7091

**Table 11:** Comparison of the models used and their prediction results on test data with SMOTE.

Model	Accuracy	Precision	Recall	F1	BACC	MCC	AUC_ROC
Random Forest	1.00	1.00	1.00	1.00	0.9998	0.9996	0.9998
AdaBoost	0.56	0.43	0.56	0.49	0.5642	0.4456	0.7095
GBT	1.00	1.00	1.00	1.00	0.9987	0.9980	0.9991
DNN	1.00	0.99	1.00	0.99	0.9975	0.9936	0.9981

**Table 12:** Comparison of the models used and their prediction results on test data without SMOTE.

Model	Accuracy	Precision	Recall	F1	BACC	MCC	AUC_ROC
Random Forest	0.99	0.99	0.99	0.99	0.9904	0.9873	0.9987
AdaBoost	0.54	0.43	0.54	0.47	0.5423	0.4190	0.7095
GBT	1.00	1.00	1.00	1.00	0.9979	0.9977	0.9990
DNN	1.00	0.99	1.00	0.99	0.9975	0.9936	0.9981

**Table 13:** Statistical analysis of the classifiers by accuracy of the model based on paired Wilcoxon test with *p*-value 0.05.

Classifiers	*p*-Value	Z-Value	W-Value	Comparison
RandomForest/GBT	0.0151	−2.4303	21	Significant at *p* < 0.05.
RandomForest/AdaBoost	0.00001	−4.7821	0	Significant at *p* < 0.05.
RandomForest/DNN	0.31732	−1.0032	136	Not significant at *p* < 0.05
AdaBoost/DNN	0.00001	−4.7821	0	Significant at *p* < 0.05.
AdaBoost/GBT	0.00001	−4.7821	0	Significant at *p* < 0.05.
DNN/GBT	0.71884	−0.362	61	Not significant at *p* < 0.05.

## Data Availability

The dataset will be published on the project website—https://simargl.eu/.

## References

[1] Zarpelão B.B., Miani R.S., Kawakani C.T., de Alvarenga S.C. (2017). A survey of intrusion detection in Internet of Things. J. Netw. Comput. Appl..

[2] Kozik R., Choraś M., Flizikowski A., Theocharidou M., Rosato V., Rome E. (2015). Advanced services for critical infrastructures protection. J. Ambient. Intell. Humaniz. Comput..

[3] Ficco M., Choraś M., Kozik R. (2017). Simulation platform for cyber-security and vulnerability analysis of critical infrastructures. J. Comput. Sci..

[4] Caviglione L., Choras M., Corona I., Janicki A., Mazurczyk W., Pawlicki M., Wasielewska K. (2021). Tight Arms Race: Overview of Current Malware Threats and Trends in Their Detection. IEEE Access.

[5] Choraś M., Pawlicki M. (2021). Intrusion detection approach based on optimised artificial neural network. Neurocomputing.

[6] Kozik R., Pawlicki M., Choraś M. (2021). A new method of hybrid time window embedding with transformer-based traffic data classification in IoT-networked environment. Pattern Anal. Appl..

[7] Dutta V., Choras M., Pawlicki M., Kozik R. (2020). A Deep Learning Ensemble for Network Anomaly and Cyber-Attack Detection. Sensors.

[8] Khraisat A., Gondal I., Vamplew P., Kamruzzaman J. (2019). Survey of intrusion detection systems: Techniques, datasets and challenges. Cybersecurity.

[9] Masdari M., Khezri H. (2020). A survey and taxonomy of the fuzzy signature-based Intrusion Detection Systems. Appl. Soft Comput..

[10] Daniya T., Suresh Kumar K., Santhosh Kumar B., Sekhar Kolli C. (2021). A survey on anomaly based intrusion detection system. Mater. Today Proc..

[11] Liu J., Gao Y., Hu F. (2021). A fast network intrusion detection system using adaptive synthetic oversampling and LightGBM. Comput. Secur..

[12] He H., Bai Y., Garcia E.A., Li S. ADASYN: Adaptive synthetic sampling approach for imbalanced learning. Proceedings of the 2008 IEEE International Joint Conference on Neural Networks (IEEE World Congress on Computational Intelligence).

[13] Ke G., Meng Q., Finley T., Wang T., Chen W., Ma W., Ye Q., Liu T.Y. (2017). Lightgbm: A highly efficient gradient boosting decision tree. Adv. Neural Inf. Process. Syst..

[14] Tavallaee M., Bagheri E., Lu W., Ghorbani A.A. A detailed analysis of the KDD CUP 99 data set. Proceedings of the 2009 IEEE Symposium on Computational Intelligence for Security and Defense Applications.

[15] Moustafa N., Slay J. UNSW-NB15: A comprehensive data set for network intrusion detection systems (UNSW-NB15 network data set). Proceedings of the 2015 Military Communications and Information Systems Conference (MilCIS).

[16] Sharafaldin I., Habibi Lashkari A., Ghorbani A.A. Toward Generating a New Intrusion Detection Dataset and Intrusion Traffic Characterization. Proceedings of the 4th International Conference on Information Systems Security and Privacy—Volume 1: ICISSP, INSTICC, SciTePress.

[17] Sohi S.M., Seifert J.P., Ganji F. (2021). RNNIDS: Enhancing network intrusion detection systems through deep learning. Comput. Secur..

[18] Mebawondu J.O., Alowolodu O.D., Mebawondu J.O., Adetunmbi A.O. (2020). Network intrusion detection system using supervised learning paradigm. Sci. Afr..

[19] Zhou Y., Cheng G., Jiang S., Dai M. (2020). Building an efficient intrusion detection system based on feature selection and ensemble classifier. Comput. Netw..

[20] Kolias C., Kambourakis G., Stavrou A., Gritzalis S. (2016). Intrusion detection in 802.11 networks: Empirical evaluation of threats and a public dataset. IEEE Commun. Surv. Tutor..

[21] García S., Grill M., Stiborek J., Zunino A. (2014). An Empirical Comparison of Botnet Detection Methods. Comput. Secur..

[22] Damasevicius R., Venckauskas A., Grigaliunas S., Toldinas J., Morkevicius N., Aleliunas T., Smuikys P. (2020). LITNET-2020: An Annotated Real-World Network Flow Dataset for Network Intrusion Detection. Electronics.

[23] McCanne S. (2011). libpcap: An Architecture and Optimization Methodology for Packet Capture. http://sharkfest.wireshark.org/sharkfest.11/presentations/McCanne-Sharkfest%2711_Keynote_Address.pdf.

[24] Okiru Malware Puts Billions of Connected Devices at Risk. https://searchsecurity.techtarget.com/news/252433491/Okiru-malware-puts-billions-of-connected-devices-at-risk.

[25] Kolias C., Kambourakis G., Stavrou A., Voas J. (2017). DDoS in the IoT: Mirai and other botnets. Computer.

[26] Alomari E., Manickam S., Gupta B., Karuppayah S., Alfaris R. (2012). Botnet-based distributed denial of service (DDoS) attacks on web servers: Classification and art. arXiv.

[27] Lee C.B., Roedel C., Silenok E. (2003). Detection and Characterization of Port Scan Attacks.

[28] Allen L., Heriyanto T., Ali S. (2014). Kali Linux—Assuring Security by Penetration Testing.

[29] Haja D., Szabo M., Szalay M., Nagy A., Kern A., Toka L., Sonkoly B. How to orchestrate a distributed OpenStack. Proceedings of the IEEE INFOCOM 2018—IEEE Conference on Computer Communications Workshops (INFOCOM WKSHPS).

[30] Tesliuk A., Bobkov S., Ilyin V., Novikov A., Poyda A., Velikhov V. Kubernetes Container Orchestration as a Framework for Flexible and Effective Scientific Data Analysis. Proceedings of the 2019 Ivannikov Ispras Open Conference (ISPRAS).

[31] Lyon G.F. (2009). Nmap Network Scanning: The Official Nmap Project Guide to Network Discovery and Security Scanning.

[32] robertdavidgraham/masscan: TCP Port Scanner, Spews SYN Packets Asynchronously, Scanning Entire Internet in under 5 Minutes. https://github.com/robertdavidgraham/masscan.

[33] CAPEC—CAPEC-287: TCP SYN Scan (Version 3.4). https://capec.mitre.org/data/definitions/287.html.

[34] Tarasov Y., Pakulova E., Basov O. (2019). Modeling of Low-Rate DDoS-Attacks. Proceedings of the 12th International Conference on Security of Information and Networks, (SIN’19).

[35] Najafabadi M.M., Khoshgoftaar T.M., Napolitano A., Wheelus C. Rudy attack: Detection at the network level and its important features. Proceedings of the Twenty-Ninth International Flairs Conference.

[36] Apache Kafka. https://kafka.apache.org/.

[37] Deri L., Martinelli M., Cardigliano A. Realtime high-speed network traffic monitoring using ntopng. Proceedings of the 28th Large Installation System Administration Conference (LISA14).

[38] Komisarek M., Pawlicki M., Kozik R., Choras M. (2021). Machine Learning Based Approach to Anomaly and Cyberattack Detection in Streamed Network Traffic Data. J. Wirel. Mob. Netw. Ubiquitous Comput. Dependable Appl..

[39] Chandrashekar G., Sahin F. (2014). A survey on feature selection methods. Comput. Electr. Eng..

[40] Longadge R., Dongre S. (2013). Class imbalance problem in data mining review. arXiv.

[41] Burduk R. (2020). Classification Performance Metric for Imbalance Data Based on Recall and Selectivity Normalized in Class Labels. arXiv.

[42] Thabtah F., Hammoud S., Kamalov F., Gonsalves A. (2020). Data imbalance in classification: Experimental evaluation. Inf. Sci..

[43] Chawla N., Bowyer K., Hall L., Kegelmeyer W. (2002). SMOTE: Synthetic Minority Over-sampling Technique. J. Artif. Intell. Res. (JAIR).

[44] Mukherjee M., Khushi M. (2021). SMOTE-ENC: A Novel SMOTE-Based Method to Generate Synthetic Data for Nominal and Continuous Features. Appl. Syst. Innov..

[45] Chicco D., Jurman G. (2020). The advantages of the Matthews correlation coefficient (MCC) over F1 score and accuracy in binary classification evaluation. BMC Genom..

[46] sklearn.feature_selection.SelectKBest —Scikit-learn 0.24.2 Documentation. https://scikit-learn.org/stable/modules/generated/sklearn.feature_selection.SelectKBest.html.

[47] McCulloch W.S., Pitts W. (1943). A logical calculus of the ideas immanent in nervous activity. Bull. Math. Biophys..

[48] Kelley H.J. (1960). Gradient theory of optimal flight paths. ARS J..

[49] LeCun Y., Bengio Y., Hinton G. (2015). Deep learning. Nature.

[50] Ho T.K. Random decision forests. Proceedings of the 3rd International Conference on Document Analysis and Recognition.

[51] Breiman L. (2001). Random forests. Mach. Learn..

[52] Freund Y., Schapire R., Abe N. (1999). A short introduction to boosting. J. Jpn. Soc. Artif. Intell..

[53] Friedman J.H. (2001). Greedy function approximation: A gradient boosting machine. Ann. Stat..

[54] Pawlicki M., Choraś M., Kozik R., Hołubowicz W. (2020). On the Impact of Network Data Balancing in Cybersecurity Applications. International Conference on Computational Science.

[55] Kozik R., Pawlicki M., Choraś M. (2018). Cost-sensitive distributed machine learning for netflow-based botnet activity detection. Secur. Commun. Netw..

[56] Kingma D.P., Ba J. (2014). Adam: A method for stochastic optimization. arXiv.

[57] Taheri S.M., Hesamian G. (2013). A generalization of the Wilcoxon signed-rank test and its applications. Stat. Pap..

